# Acute Myocardial Infarction as the First Presentation of Systemic Lupus Erythematosus in a 23-Year-Old Patient

**DOI:** 10.7759/cureus.41026

**Published:** 2023-06-27

**Authors:** Mohammed Ayyad, Maram Albandak, Mansour Khaleel, Nabil C N Khalil, Muaath Itmaizeh

**Affiliations:** 1 Internal Medicine, Al-Quds University, Jerusalem, PSE; 2 Internal Medicine, Al-Makassed Charitable Society Hospital, Jerusalem, PSE; 3 Rheumatology, Al-Makassed Charitable Society Hospital, Jerusalem, PSE

**Keywords:** antinuclear antibodies (ana), anti-double-stranded dna antibodies (anti-dsdna), autoimmune disease, dual-antiplatelet therapy (dapt), primary pci, stemi, acute coronary syndrome, atherosclerosis, systemic lupus erythematosus, myocardial infarction

## Abstract

ST-segment elevation myocardial infarction (STEMI) in young adults is a rare occurrence that requires a thorough investigation to determine the underlying cause. Herein, a young female patient presented with dull retrosternal chest pain associated with nausea and left arm numbness. Cardiac-specific troponin was elevated and the electrocardiogram revealed ST-segment elevation in the inferior wall leads indicative of myocardial infarction. The patient was started on dual antiplatelet therapy (DAPT) and emergency coronary angiography was performed, revealing a 20% stenosis in the left circumflex artery and evidence of a thrombotic lesion in the posterolateral branch (PLB), which was deemed unsuitable for intervention. During the diagnostic workup, the patient tested positive for antinuclear antibodies and was ultimately diagnosed with systemic lupus erythematosus (SLE) and antiphospholipid syndrome.

This case highlights the rarity of STEMI as an initial presentation of SLE. It emphasizes the importance of considering autoimmune disorders in young patients with acute myocardial infarction and the need for a comprehensive evaluation and appropriate management in such cases.

## Introduction

Systemic lupus erythematosus (SLE) is a chronic inflammatory disease of unknown cause that can affect the skin, joints, lungs, nervous system, serous membranes, and/or other organs of the body [1]. SLE has been associated with an increased risk of cardiovascular disease, including ischemic heart disease (IHD), which affects up to 16% of patients with the condition [2]. Among patients with SLE, IHD is commonly attributed to atherosclerosis, which is more prevalent due to systemic inflammation and the adverse effects of long-term glucocorticoid treatment [1]. However, the initial manifestations are usually non-cardiac symptoms and include arthralgias, arthritis, and/or skin involvement. While tamponade or myopericarditis can be the initial cardiac presentation, a few cases have reported ST-segment elevation acute myocardial infarction (STEMI) as the initial manifestation [3-7]. In this case report, we present the case of a 23-year-old female who experienced acute myocardial infarction as the initial presentation of SLE.

## Case presentation

A 23-year-old female patient presented to the hospital with retrosternal chest pain described as dullness. The chest pain was not associated with physical exertion; however, it was relieved by the administration of sublingual nitroglycerin and exacerbated by deep breathing. The patient also reported experiencing severe nausea and numbness in her left forearm. There was no history of trauma, cough, fever, syncope, or palpitations. Her medical history was significant for hypothyroidism well-controlled on levothyroxine. Past surgical history was notable for a recent spontaneous abortion at a gestational age of two months. Further questioning revealed no history of alcohol consumption, smoking, or obesity (BMI=20.2). Additionally, there was a family history of sudden cardiac death in her father at the age of 52, but no known family history of autoimmune disease.

On examination, the patient was acutely distressed due to pain. Vital signs revealed a heart rate of 68 bpm, a blood pressure of 120/73 mm Hg, an oxygen saturation (SaO2) of 99% on room air, and a temperature of 37 °C. Cardiac examination revealed a normal S1 and S2, with no extra sounds such as a friction rub, gallops, or murmurs. The lung examination revealed bilateral vesicular breath sounds with no crackles or wheezing. On neck examination, the jugular venous pressure was normal with an estimated venous pressure of 7 cm of water. Chest wall palpation revealed no chest wall tenderness. Moreover, the pain did not show obvious exacerbation or relief with any change in body position.

**Figure 1:**
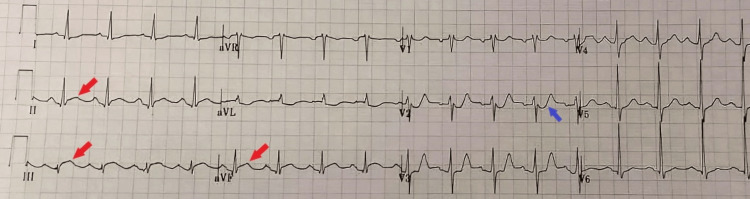
12-lead ECG findings on admission showing ST-segment elevation of the inferior leads (red arrows) accompanied by reciprocal changes (blue arrow).

Laboratory investigations including a complete blood count (CBC), basic metabolic panel, and kidney function tests were all normal. The troponin level on admission was 1.619 ng/mL and the erythrocyte sedimentation rate was elevated at 63 mm/hr (0-20 mm/hr). An electrocardiogram (ECG) revealed ST-segment elevation of the inferior leads in association with reciprocal changes most prominent in lead V2 (Figure 1). Echocardiogram showed hypokinesia in the inferior part of the interventricular septum. The left ventricular function was normal with an ejection fraction of 55%. Moreover, there were no signs of an increased pericardial thickness or pericardial effusion. Subsequently, the patient was started on dual antiplatelet therapy, morphine, and nitroglycerin.

**Figure 2:**
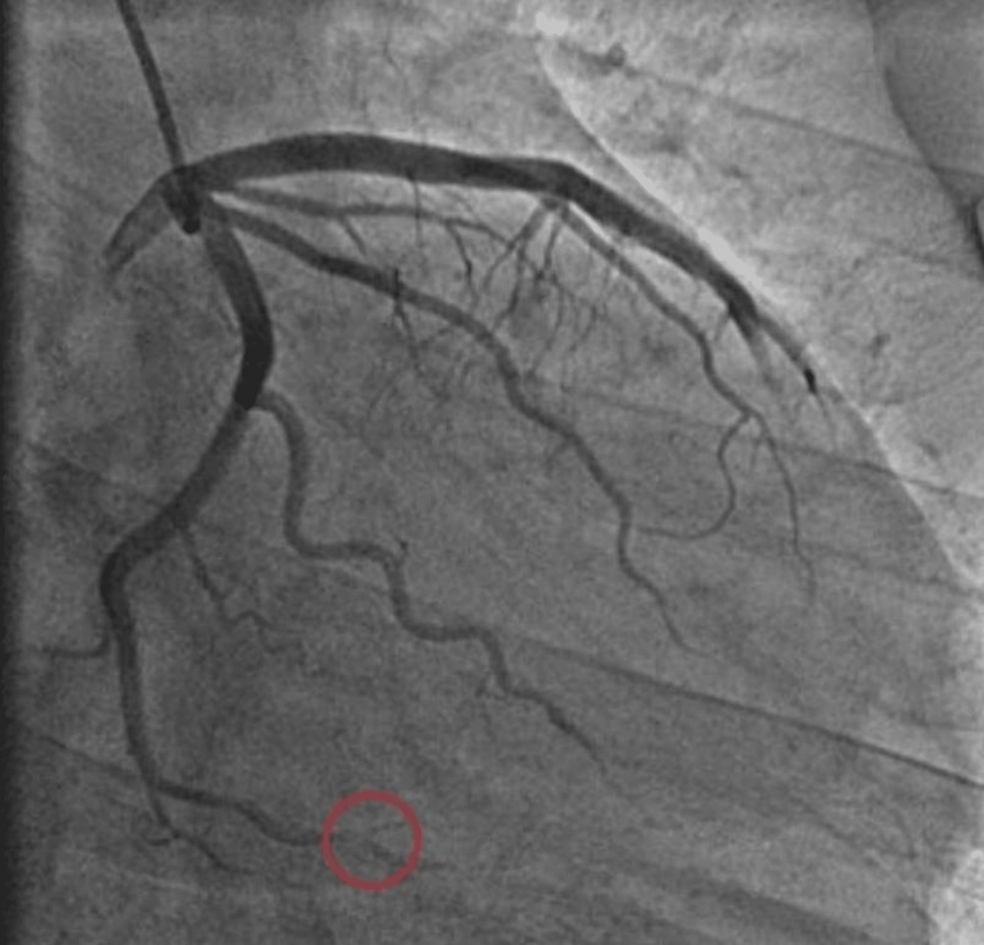
Cardiac catheterization findings in our patient using the right radial approach. The angiogram shows distal slow flow in the posterolateral branch (PLB) of the right coronary artery, indicative of possible thrombus formation (red circle). The small caliber of the vessel makes it unsuitable for immediate intervention.

Cardiac catheterization was performed using the right radial approach, which revealed 20% stenosis in the left circumflex artery and evidence of a thrombotic lesion in the posterolateral branch (PLB) (Figure 2). The results suggested the presence of a thrombus which was not suitable for intervention due to its small caliber. Following the catheterization, dual antiplatelet therapy (DAPT) was continued and the patient was started on high-dose atorvastatin.

After appropriate treatment, serial troponin levels were ordered and revealed a downward trend in enzyme levels. Further workup to detect the underlying etiology revealed thrombocytopenia with a platelet count of 98,000 platelets per microliter, and anemia with a hemoglobin of 9.9 grams per deciliter (g/dL) (12.5-14.5 g/dL). Coagulation studies revealed a prothrombin time (PT) of 14.3 seconds (11-15 seconds), and an elevated partial thromboplastin time (PTT) at 64.5 seconds (24-36 seconds). Furthermore, the peripheral blood smear showed normocytic normochromic anemia associated with lymphocytosis, raising suspicion for an underlying rheumatological disease. Subsequently, a comprehensive rheumatologic workup was performed revealing a normal C3 level of 91 mg/dL (88-206 mg/dL), and a low C4 level of 11 mg/dL (16-48 mg/dL). Additionally, the patient tested positive for antiphospholipid antibodies, including positive lupus anti-coagulant, positive anticardiolipin antibody 27 (N<12.5 MPL U/mL), positive anti-beta 2 glycoprotein IgM antibody 21 (N<5 MPL U/mL), positive antinuclear antibodies ELISA screen 1.7 U (normal less than 1.2 U) and positive anti-double stranded DNA (dsDNA) antibodies 68 mg/dL (normal less than 30 mg/dL), all of which were consistent with the diagnosis of SLE associated with antiphospholipid syndrome. The patient was continued on medical management for acute coronary syndrome and was started on hydroxychloroquine to control her autoimmune disorder.

To further assess the possibility of a patent foramen ovale (PFO), a bubble study was performed and was unrevealing. The transesophageal echocardiography results indicated no signs of endocarditis or thrombi in the left atrial appendage and no evidence of an atrial septal defect (ASD) or PFO.

## Discussion

SLE is an autoimmune disease characterized by a multisystemic immuno-inflammatory process. This condition has been associated with a hypercoagulable state related to chronic inflammation, resulting in narrowing of the vascular lumen as well as hyperviscosity facilitating the development of thrombotic events [8]. Additionally, SLE has been associated with the development of atherosclerosis. This is theorized to be attributed to a chronic immunoinflammatory process that includes excessive cytokine production, autoantibody deposition, and complement activation, all of which culminate in accelerated atheroma formation in long-standing SLE patients [9]. Interestingly, our patient developed an acute myocardial infarction (AMI) as the first presentation of SLE, which is very unusual. One previous study describing a large cohort of SLE patients demonstrated that the average age of developing AMI in this population was 69 years of age. Furthermore, the patients were followed up to 20 years after establishing the diagnosis of SLE to assess cardiovascular morbidity and mortality [10].

Although the detection of AMI is clinically unchallenging, the development of this condition in the younger population should warrant a comprehensive diagnostic evaluation for an underlying etiology. This includes performing radiological, infectious, neoplastic, illicit drug use, and rheumatologic workups [11]. While AMI is most commonly a late presentation of SLE, it can rarely present early in the course of the disease, possibly because AMI in these patients could occur without evidence of coronary atherosclerosis. The pathophysiology of non-atherosclerotic AMI in SLE patients includes myocarditis, coronary arteritis, coronary thrombosis, and coronary artery embolization with spontaneous recanalization [12]. These non-atherosclerotic variations can create a diagnostic dilemma as they show an absence of atherosclerotic plaque on coronary angiography. However, the use of endomyocardial biopsy and/or echocardiography can be valuable in identifying the underlying cause [2].

Clinically, the first presentation of SLE usually involves constitutional symptoms as well as musculoskeletal and mucocutaneous manifestations, with cardiovascular involvement occurring rarely [13]. Nonetheless, the constellation of signs and symptoms related to SLE as well as laboratory investigations plays a role in early detection. Prompt diagnosis is crucial as the initiation of immunosuppressive therapy dampens the immunoinflammatory process causing the arteritis, which is often needed to adequately treat the AMI in this population alongside the standard therapy [2].

Interestingly, a link between anti-dsDNA antibodies and the development of cardiovascular disease in SLE patients has been established. This is postulated to be caused by alteration of molecular processes leading to a distinctive immunoinflammatory process. This process is associated with higher levels of circulating proinflammatory markers and oxidative stress molecules triggering a coagulopathic state. It’s also associated with direct endothelial injury, accelerated atheroma formation, and dysfunctional alteration of gene expression in multiple cell lineages [14]. This association could aid in early stratification of cardiovascular risk in SLE patients based on the presence and absence of anti-dsDNA and the magnitude of its titer, which could facilitate the implementation of cardioprotective measures as well as the initiation of more aggressive immunosuppressive therapy in high-risk patients.

## Conclusions

This case highlights the rare but significant presentation of AMI as the initial presentation of SLE in a young patient. The atypical cardiac manifestation underscores the importance of considering SLE in the differential diagnosis during the evaluation of acute cardiovascular events, especially in the younger population. Early recognition of this association can facilitate timely diagnosis, appropriate risk stratification, and the implementation of proactive measures to manage both AMI and underlying SLE. Further studies are warranted to better understand the underlying mechanisms and establish optimal management strategies for this patient population.
